# Reviewed article: Micheletti VCD, Rosa RS, Dipp T, Silva LP, Campagnolo PDB, Mercaus LR, Brito JR, Bonin MEM. Cardiovascular risk and statin prescription in primary care: an observational study, São Leopoldo, 2018-2021. Epidemiol Serv Saude. 2025:34;e20240432

**DOI:** 10.1590/S2237-96222025v34e20240432.b

**Published:** 2025-10-20

**Authors:** Thayssa Maluff de Mello

**Affiliations:** 1 Universidade Federal de Mato Grosso do Sul, Campo Grande, MS, Brazil

## Peer review B

## Questionnaire

Does the manuscript contain new and significant information to justify publication? Yes

Does the Abstract (Summary) clearly and accurately describe the content of the article? No

Is the problem significant and concisely stated? No

Are the methods described comprehensively? No

Are the interpretations and conclusions justified by the results? No

Is adequate reference made to other work in the field? Yes

## Manuscript Structure

Length of article is: Adequate

Number of tables is: Adequate

Number of figures is: Too few

Please state any conflict(s) of interest that you have in relation to the review of this paper. None

Interest: Good

Quality: Average

Originality: Average

Overall: Average

Recommendation: Minor Revision

## Comments

Traga o nome da cidade da Região Metropolitana de Porto Alegre. Essa recomendação destaca-se por estar o manual do Autor que no título do manuscrito deve-se informar o tema principal, delineamento, local e ano (s) da pesquisa, em consonância com o guia de redação aplicável. Lembrando que siglas não são aceitas

em títulos e UF deve ser grafada por extenso.

